# An unusual presentation of a rare disease: acute upper limb ischemia as the presenting symptom of Whipple’s Endocarditis, a case report

**DOI:** 10.1186/s12879-023-08148-5

**Published:** 2023-03-27

**Authors:** York Chen, Rattanaporn Mahatanan, Isabella W. Martin, David de Gijsel

**Affiliations:** Department of Medicine, Dartmouth-Hitchcock Medical Center, 1 Medical Center Dr, Lebanon, NH 03766 USA

**Keywords:** Whipple’s disease, Endocarditis, Embolization, Acute limb ischemia

## Abstract

**Background:**

Whipple's disease is known to cause multiple varied systemic symptoms, and is a well-documented cause of culture-negative endocarditis. Endocarditis secondary to Whipple disease, however, has rarely been known to present primarily as a cause of acute limb ischemia. We describe such a case here.

**Case presentation:**

A previously healthy 40 year old man presented to the emergency department with acute-onset right arm paresthesias. On exam, he was found to be tachycardic with a VI/VI systolic ejection murmur. He was diagnosed with critical limb ischemia and severe aortic regurgitation, and echocardiography showed a large mass on his bicuspid aortic valve. Thrombectomy was performed urgently, with aortic valve repair the following day. As blood cultures and valvular tissue culture remained unrevealing, the patient remained on empiric vancomycin and ceftriaxone for culture-negative endocarditis. 16 s rRNA nucleic acid amplification testing (NAAT) of his formalin-fixed, paraffin-embedded valvular tissue detected *T. whipplei*, after which the patient was transitioned to ceftriaxone and trimethoprim-sulfamethoxazole for a year of therapy. He continues to do clinically well.

**Conclusions:**

We report an unusual presentation of Whipple endocarditis as an acute upper limb ischemia, absent other classic symptoms of Whipple's disease, and with diagnosis made by 16 s rRNA NAAT of valvular tissue in the setting of culture-negative endocarditis.

## Background

Whipple’s disease is a rare disease caused by *Tropheryma whipplei*, a gram-positive bacillus. The spectrum of clinical findings due to *T. whipplei* infection is wide [1, 2]. Classic Whipple’s disease involves multiple organ systems and causes a well-described syndrome of gastrointestinal symptoms, arthralgia, and weight loss. A positive periodic acid-Schiff (PAS) stain on a histopathologic specimen is the gold standard for the diagnosis of Whipple’s disease; however, targeted nucleic acid amplification testing (NAAT) of various tissues has recently been more widely used for diagnosis [3, 4]. *T. whipplei* is also known as an uncommon cause of blood culture-negative endocarditis (BCNE). The diagnosis of *T. whipplei* endocarditis is challenging, with few cases reported in the literature [5]. The clinical presentation of *T. whipplei* endocarditis can vary; the majority of patients present without fever or typical manifestations of Whipple’s disease [6, 7]. Here, we report a case of *T. whipplei* endocarditis presenting with acute limb ischemia due to an acute arterial thrombus absent other overt symptoms more often described with Whipple’s disease.

## Case presentation

A 40-year-old man with no significant past medical history except tobacco use presented to the emergency department (ED) at a local hospital with right arm paresthesia and worsening pain for two days. He reported lethargy over the preceding few months and night chills but no measured fevers. He recalled a transient episode of diarrhea several weeks prior, at which point he felt moderately fatigued. He fully recovered from this episode without seeking medical care. On initial exam, the patient was afebrile but tachycardic with a heart rate of 135 beats/min. A VI/VI systolic cardiac murmur was present. His right radial pulse was undetectable both manually and by Doppler sonography. A CT angiogram of the right arm showed evidence of an occlusive thrombus in his right brachial artery. The patient was started on full dose therapeutic anticoagulation with a heparin drip and was transferred to our hospital for management of the acute arterial occlusion.

**Fig. 1:**
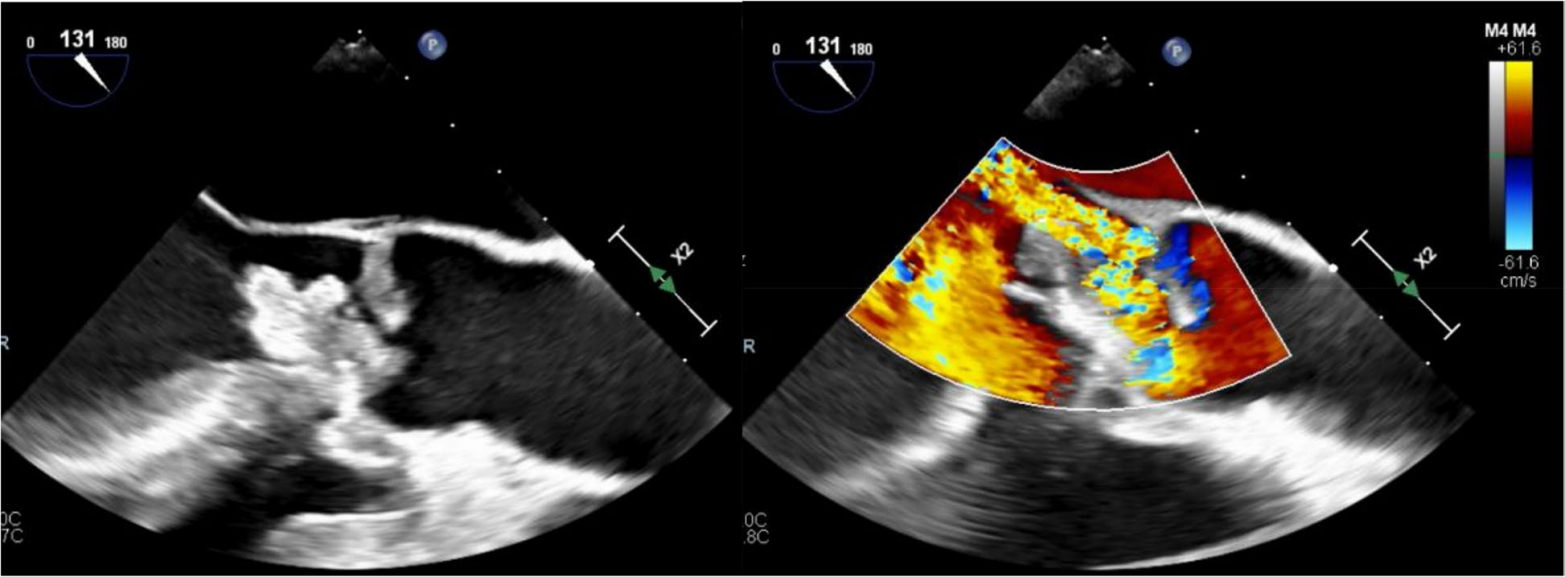
Transesophageal echocardiography findings. Still image on the left demonstrating a large mobile vegetation on his bicuspid aortic valve, while the image on the right is a still Doppler image showing significant aortic regurgitation. Images provided by Ethan Senser, MD

On the day of transfer, he underwent right brachial artery cutdown and thrombectomy of the axillary, brachial, radial, and ulnar arteries. Intra-operatively, an inflamed brachial artery was seen and a large volume of white embolic debris with mixed acute thrombus was removed and submitted for aerobic and anaerobic bacterial culture. The Gram stain showed many neutrophils without organisms. Intact distal flow and Doppler signals were seen in both radial and ulnar arteries post-op. A post-operative bedside echocardiogram showed severe aortic regurgitation. A formal transthoracic echocardiogram revealed an irregularly shaped, multilobar mobile mass measuring greater than 2.5 cm on the aortic valve with severe 4 + /4 + aortic valve regurgitation; this was corroborated on transesophageal echocardiogram (Fig. 1). He developed a fever of 101.7 F (38.7 C). He had a white blood cell (WBC) count of 18,200 /mm^3^ on admission, and a basic metabolic panel and liver function test within the normal range. He was empirically given vancomycin and ceftriaxone after blood cultures were drawn.

**Fig. 2:**
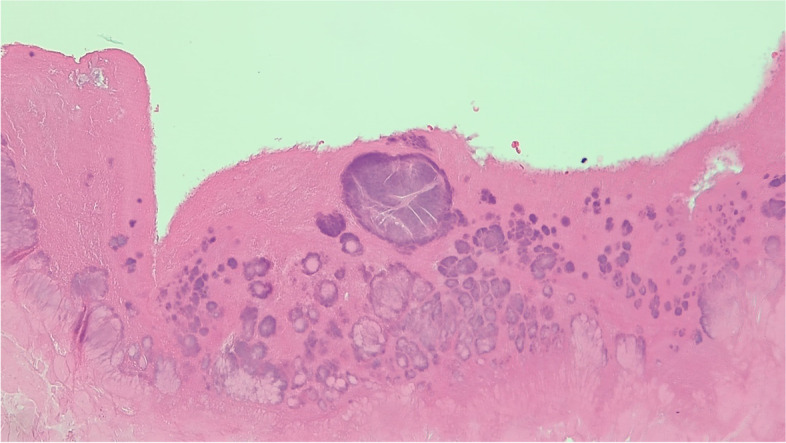
Hematoxylin & eosin stain at 1000 × magnification of aortic valve vegetation showing extensive necrosis and fibrin-rich material with aggregates of coccoid bacteria

One day after admission, within 24 h after starting antibiotics, he was taken to the operating room for an aortic valve replacement with a 23 mm bovine valve. Intra-operatively, his aortic valve appeared bicuspid, although no clearly identifiable native tissue remained due to valvular destruction. There was also a significant paravalvular leak. Aortic valvular tissue submitted for aerobic and anaerobic culture showed no organisms or neutrophils on Gram stain and had no growth after four days. Histologic examination of the valvular tissue showed extensive necrosis and fibrin-rich vegetations with bacterial forms present (Fig. 2). In total, 10 blood cultures were drawn with no growth.

The patient quickly defervesced on vancomycin and ceftriaxone, with interval improvement of leukocytosis without resolution. The patient continued to recover post-operatively with intact right upper extremity neurovascular recovery, and his heparin drip was quickly discontinued in light of known removal of his embolic source on hospital day 2. Physical exam showed no other evidence of embolic phenomena. Full mouth dental extraction was pursued given his extensive dental caries and new bioprosthetic valve. No further imaging workup was pursued. A timeline of relevant clinical events and data points follows (Table 1).

**Table 1:** Select hospital days with sequence of clinical events and antibiotic course

Day 0	WBC 18.2, Tmax 38.7 C admitted for acute limb ischemia with heparin drip started and taken to OR for thrombectomy 4 blood cultures negative antibiotics initiated: vancomycin and piperacillin-tazobactam
Day 1	Bovine prosthetic aortic valve placed; aortic valvular tissue sent for aerobic, anaerobic, and fungal culture 2 blood cultures negative antibiotics transitioned to vancomycin and ceftriaxone
Day 2	WBC 5.2, Tmax 36.7 therapeutic anticoagulation discontinued 2 blood cultures negative; antibiotics continued
Day 3	WBC 13.6 Tmax 36.8 2 blood cultures negative; antibiotics continued consultation to OMFS for dental extraction as possible source
Day 6	WBC 14.2 Tmax 36.7 Full dental extraction of 28 teeth (1,2,3,4,5,6,7,9,10,11,12,13,14,17, 19,20,21,22,23,24,25,26,27,28,29,30,31,32) antibiotics continued

Additional laboratory work-up for blood culture-negative endocarditis was initiated, including urine antigen testing for *Legionella pneumophila* and serum antibody testing for *Bartonella* spp. and *Mycoplasma pneumoniae*, all of which were negative. Without travel or obvious farm animal exposure, serologic testing for *Brucella melitensis* and *Coxiella burnetii* was deferred. Given the negative tissue culture results and the visualization of microorganisms on histologic examination (Fig. 2), the formalin-fixed, paraffin-embedded (FFPE) valvular tissue was sent to a referral laboratory for amplification and sequencing of a highly variable fragment of the bacterial 16 s ribosomal RNA gene (“broad-range bacterial NAAT”) [8].

On discharge a C-reactive protein resulted at 130 mg/L The patient was discharged with outpatient parenteral vancomycin and ceftriaxone therapy for 6 weeks. After discharge, the broad-range bacterial NAAT returned with detection of *T. whipplei.* Vancomycin was discontinued at that point after 2 weeks of therapy, IV ceftriaxone 2 g daily was continued to complete a 4-week course, followed by oral trimethoprim-sulfamethoxazole 800–160 mg BID to complete 12 months of treatment. The patient tolerated therapy well at a 12-month follow up. A duodenal biopsy with PAS stain confirmed absence of *T. whipplei*.

## Discussion and conclusions

*Tropheryma whipplei*, the causative organism in Whipple’s disease, is a Gram-positive bacillus classically causing chronic diarrhea leading to malnutrition. Clinically, the disease can present in many different ways, including polyarthralgias, endocarditis, and neuropsychiatric changes.

Whipple’s endocarditis is a rare disease in the literature, and is on the differential for BCNEThe presentation of Whipple’s endocarditis ranges from arthralgia to heart failure. A 2012 publication by Geissdorfer et al. found *T. whipplei* infection in 6% of all bacterial endocarditis cases, confirmed by specific PCR and culture [9]. In one large case series from 2001 encompassing 35 patients with Whipple’s endocarditis, 89% were male with the majority of patients being afebrile and not meeting Duke criteria for endocarditis [5, 6, 10]. Commonly, aortic and/or mitral valves are involved [6, 7].

Our patient presented with an acute peripheral arterial occlusion likely from a migrated cardiac vegetation due to Whipple’s endocarditis. Septic embolization is a common complication of infectious endocarditis (IE), and systemic embolization most commonly occurs in left-sided IE, potentially causing stroke, blindness, or splenic infarction [11–13]. The complication of acute limb ischemia from endocarditis remains uncommon. In a cohort of patients with IE, Uglov et al. report only 4.5% (12 out of 265 patients) presenting with thromboembolism of the arteries of the limbs. The majority of these patients required surgical interventions [14, 15]. Given the rarity of Whipple’s endocarditis, the occurrence of systemic embolization in this infection in the literature is extremely rare. Table 2 details four such documented cases.

**Table 2:** A list of four documented case reports of Whipple’s endocarditis causing embolism, all with valvular replacement and valve tissue NAAT diagnosis

Citation	Patient	Microbiology Diagnostics	Treatment and Outcome
Naegeli 2000 [16]	51 year old woman with recurrent strokes, found to have mitral valve involvement	PAS positive valve histology confirmed with unspecified NAAT of valve tissue	TMP-SMX with clinical improvement
Richardson 2003 [17]	51 year old man with right axillary embolism, found to have aortic valve vegetations	Aortic valve broad-range bacterial NAAT	4 weeks of vancomycin followed by 12 months of TMP-SMX with clinical improvement
Seddon 2017 [18]	54 year old woman with ulnar artery embolism, found to have aortic valve vegetations	Aortic valve broad-range bacterial NAAT	6 weeks of empiric amoxicillin and gentamicin with clinical improvement
He 2021 [19]	53 year old man with right calf pain and popliteal, tibial-peroneal trunk, and tibial artery clot, found to have aortic valve vegetations	PAS positive valve histology confirmed with broad-range bacterial NAAT of valve tissue	Ceftriaxone 2 g daily for 6 weeks, followed by TMP-SMX double-strength two tablets twice daily for 18 months with clinical improvement

Blood cultures obtained before the administration of antimicrobial therapy remain the mainstay of organism identification in the pre-operative diagnosis of infective endocarditis. In the absence of blood culture growth, optimal routine evaluation of excised cardiac valvular tissue includes both bacterial culture and histologic examination. Due to *T. whipplei*’s extremely slow-growing and fastidious nature which prevents growth in routine cultures, the diagnosis of Whipple’s endocarditis is challenging and often delayed. While a positive PAS stain of valvular tissue is considered the gold standard in the diagnosis of Whipple’s endocarditis, it has long been thought that this methodology leads to underdiagnosis. This is supported by the increased detection of *T. whipplei* in tissue by NAAT, compared to tissue PAS stain [3, 4]. The review of the literature by McGee et al. encompassing 156 cases of Whipple’s endocarditis diagnosed by direct examination of valvular tissue reports 51% case positivity by immunohistochemical staining and 72% by NAAT, compared with only 39% by PAS stain [10]. Our patient’s diagnosis was made with broad-range bacterial NAAT of FFPE valvular tissue.

Although no relevant FDA-cleared diagnostic assays exist, molecular detection of *T. whipplei* can be accomplished through laboratory-developed testing in larger laboratories or referral laboratories. Multiple diagnostic approaches can be found, including *T. whipplei*-specific NAAT, broad-range bacterial NAAT, or unbiased metagenomic NAAT assays. Depending on the laboratory and assay, testing can be performed on either a peripheral blood specimen or on excised valvular tissue, the latter using either fresh/refrigerated/frozen tissue or FFPE tissue. The sensitivity of *T. whipplei*-specific NAAT performed on peripheral blood specimens is lower than that of direct testing of valvular tissue [4, 20]. Broad-range bacterial NAAT of either fresh or FFPE valvular tissue should be considered in culture-negative cases if histopathology of resected valvular tissue demonstrates inflammatory changes and/or visible microorganisms. However, it is important to note the yield of targeted *T. whipplei*-specific NAAT tends to demonstrate greater sensitivity than broad-range bacterial NAAT for the diagnosis of Whipple’s endocarditis [4].

Whipple’s disease has a high mortality and a high relapse rate, with one case series reporting 24% mortality among 169 patients [10]. The fatality rate is difficult to determine due to the rarity of the disease and the difficulty in diagnosis. Treatment for Whipple’s disease is generally prolonged, with up to 12 months of antibiotics. The treatment includes an initial course of ceftriaxone 2 g IV for 2 weeks followed by trimethoprim-sulfamethoxazole orally for up to one year, with doxycycline and hydroxychloroquine as second-line treatments instead of trimethoprim-sulfamethoxazole [21]. Our patient completed a treatment course of IV therapy and continues on oral trimethoprim-sulfamethoxazole. He is tolerating the oral antibiotics well, without recurrence of symptoms at a 12-month follow up.

In summary, we report a case of Whipple’s endocarditis presenting with acute limb ischemia necessitating an emergent vascular intervention. The patient eventually underwent valvular replacement, and *T. whipplei* was detected by broad-range bacterial NAAT from valvular tissue, confirming the diagnosis of Whipple’s endocarditis without serology testing. This case emphasizes one of the myriad possible clinical presentations of Whipple’s endocarditis and the importance of tissue NAAT in the diagnostic workup of BCNE.

## Data Availability

Data sharing is not applicable to this article as no datasets were generated or analyzed during the current study.

## Abbreviations

BCNE: Blood culture negative endocarditis
IE: Infective endocarditis
NAAT: Nucleic acid amplification test
PAS: Periodic acid Schiff
RNA NAAT: Ribonucleic acid nucleic acid amplification test
TEE: Transthoracic echocardiogram
TMP-SMX: Trimethoprim-sulfamethoxazole
